# Initial therapeutic dose of corticosteroid for an acute exacerbation of IPF is associated with subsequent early recurrence of another exacerbation

**DOI:** 10.1038/s41598-021-85234-1

**Published:** 2021-03-11

**Authors:** Ryo Yamazaki, Osamu Nishiyama, Sho Saeki, Hiroyuki Sano, Takashi Iwanaga, Yuji Tohda

**Affiliations:** Department of Respiratory Medicine and Allergology, Kindai University Faculty of Medicine, Osakasayama, Osaka 589-8511 Japan

**Keywords:** Medical research, Risk factors

## Abstract

Some patients with idiopathic pulmonary fibrosis (IPF) undergo recurrent acute exacerbations (AEs). This study aimed to elucidate the risk factors for recurrent AEs of IPF (AE-IPF). Consecutive patients with IPF admitted for their first AE-IPF between January 2008 and December 2018 were retrospectively recruited. Of 63 patients admitted for an AE-IPF and discharged alive, 9 (14.3%) developed a recurrence of AE within 1 year. The mean time to recurrence was 233 ± 103 days. Total doses (mg/month and mg/kg/month) of corticosteroids administered over day 1 to 30 after the AE were significantly higher in patients without recurrences of AE-IPF (5185 ± 2414 mg/month, 93.5 ± 44.0 mg/kg/month) than the doses in patients with recurrences (3133 ± 1990 mg/month, 57.2 ± 37.7 mg/kg/month) (*p* = 0.02 and *p* = 0.03, respectively). However, no differences were observed between the total doses of corticosteroids administered over days 31 to 60, 61 to 90, 91 to 120, and 151 to 180 after the AE. Furthermore, differences between the administration rates of immunosuppressive and antifibrotic treatments administered to the 2 patient groups were not significant. An increased total dose of corticosteroid administered over day 1 to 30 after an AE-IPF was associated with a decreased risk of subsequent recurrence of AE-IPF within 1 year after the first AE.

## Introduction

Idiopathic pulmonary fibrosis (IPF) is a chronic, progressive, fibrosing interstitial pneumonia of unknown etiology^1^. Over their clinical course, some IPF patients undergo rapid worsening of respiratory symptoms associated with new ground-glass opacities or consolidations on chest high-resolution computed tomography (HRCT). The event is termed “acute exacerbation (AE) of IPF” (AE-IPF), and its mortality rate is high^1,2^. The treatment for AE-IPF generally consists of high-dose corticosteroids and/or immunosuppressive agents, although there have been no supportive data from randomized controlled trials^3,4^. There is also no proven specific maintenance treatment after the initial treatment.

AE-IPF sometimes recurs, even after it has resolved. A previous history of AE was reported to be associated with an increased risk of recurrence^5^. However, specific risk factors for the subsequent recurrence of an AE in patients after their first AE remain unknown. The aim of this study was to test the hypothesis that as one of the treatments, the initial dosage of corticosteroid for the first AE is associated with early recurrence of an AE-IPF.

## Results

**Figure 1:**
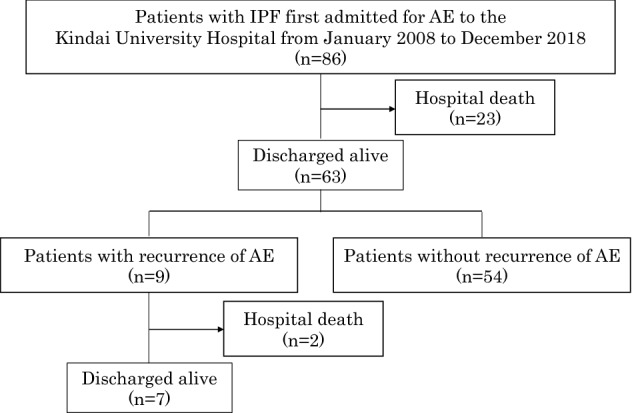
Flowchart of distribution of study patients over the period of observation. AE: acute exacerbation, IPF: idiopathic pulmonary fibrosis.

During the study period, 86 patients with IPF were hospitalized for AE (Fig. 1). Among the 86 patients, 23 (26.7%) died in the hospital and 63 (73.3%) were discharged alive. Of the 63 who were discharged, 9 (14.3%) developed a recurrent AE-IPF. The mean time from diagnosis of the first AE-IPF to recurrence was 233 ± 103 days. Among the 9 patients, 2 (22.2%) died in the hospital and 7 (77.8%) were discharged alive. Of the 7 discharged patients, 3 (42.9%) developed a second recurrence.

The baseline clinical characteristics and treatments of the 63 patients admitted for the first time and discharged alive are shown in Table 1. The differences between clinical characteristics and treatments for patients with and without recurrence of AE-IPF within 1 year after the first AE were not significant. The differences between the laboratory findings at the time of discharge after the first admission for AE-IPF for the patients with and without subsequent recurrence were not significant (Table 2). The mean daily dose of corticosteroids which each patient was taking at the time of recurrence of an AE-IPF was 7.7 ± 7.0 mg.

**Table 1:** Baseline characteristics of patients with IPF and treatment of IPF at discharge of the first acute exacerbation.

Characteristics	Patients with recurrence of AE n = 9	Patients without recurrence of AE n = 54	*p* Value
Age, yr	75.2 ± 4.4	74.6 ± 6.4	0.79
**Sex**			
Male/female	9/0	41/13	0.18
Body mass index	23.3 ± 2.7	21.6 ± 2.7^a^	0.09
**Smoking status**			
Yes/no	8/1	41/13	0.66
**Long-term oxygen therapy**			
Yes/no	4/9	21/33	0.75
Antifibrotic agent	2	3	0.14
pirfenidone/nintedanib	2/0	2/1	
**Comorbidities**			
Hypertension	5	29	> 0.99
Diabetes mellitus	2	21	0.46
Dyslipidemia	2	16	> 0.99
Atrial fibrillation/flutter	0	4	> 0.99
Coronary artery disease	1	10	> 0.99

Values are shown as actual numbers or means ± standard deviation.

^a^n = 45.

*AE* acute exacerbation, *DLco* diffusing capacity of lungs for carbon monoxide, *FEV*_*1*_ forced expiratory volume in 1 s, *FVC* forced vital capacity, *IPF* idiopathic pulmonary fibrosis.

**Table 2:** Laboratory parameters at discharge for the first acute exacerbation.

Variables	Patient with recurrence of AE n = 9	Patient without recurrence of AE n = 54	*p* Value
CRP, mg/dL	0.7 ± 1.1	0.4 ± 0.7	0.31
WBC, /µL	9077 ± 2750	8857 ± 3403	0.85
LDH, IU/L	282 ± 70	256 ± 60	0.25
Platelet counts, × 10^4^/µL	19.6 ± 9.2	21.2 ± 7.2	0.54
KL-6, U/mL	1482 ± 557	1418 ± 824	0.82
**Arterial blood gas test**			
pH	7.42 ± 0.02	7.42 ± 0.03	0.76
PaCO_2_	36.4 ± 5.1	38.8 ± 5.7	0.22
PaO_2_	79.9 ± 10.5	77.3 ± 10.4	0.48
PaO_2_/FiO_2_ ratio	339 ± 104	351 ± 55	0.59
HCO_3_^−^	23.3 ± 3.3	24.7 ± 3.6	0.27

Values are shown as actual numbers or means ± standard deviation.

*AE* acute exacerbation, *CRP* c-reactive protein, *KL-6* Krebs von der lungen-6, *LDH* lactate dehydrogenase, *PaO*_*2*_*/FiO*_*2*_ partial pressure of atrial oxygen/fraction of inspiratory oxygen, *WBC* white blood cells.

Treatments for the first AE-IPF are shown in Table 3. Corticosteroids were administered to every patient who was admitted because of an AE-IPF. Total doses (mg/month and mg/kg/month) of corticosteroids administered over days 1 to 30 after the diagnosis of AE were significantly higher in patients without recurrences of AE-IPF (5185 ± 2414 mg/month, 93.5 ± 44.0 mg/kg/month) than the doses in patients with recurrences (3133 ± 1990 mg/month, 57.2 ± 37.7 mg/kg/month) (*p* = 0.02 and *p* = 0.03, respectively). However, no differences were observed between the total doses of corticosteroids administered over days 31 to 60, 61 to 90, 91 to 120, and 151 to 180 after the AE. Furthermore, differences between the rates of administration of immunosuppressive and antifibrotic treatments to the 2 patient groups were not significant. A multivariable analysis of the following independent variables: total doses (mg/month and mg/kg/month) of corticosteroids over days 1 to 30 after the diagnosis of AE, total doses (mg/month) of corticosteroids over days 61 to 90, sex, age, BMI, and use of antifibrotic agent at discharge; identified total doses (mg/month) of corticosteroids over days 1 to 30 as the only independent predictor of recurrence (odds ratio per 1 g increase in corticosteroid dose: 0.61, 95% confidence interval: 0.41–0.90, *p* = 0.02).

**Table 3:** Therapies after onset of the first acute exacerbation in patients with and without subsequent recurrence of acute exacerbation.

Therapy	Patients with recurrence of AE n = 9	Patients without recurrence of AE n = 54	*p* Value
**Total dose of corticosteroid over**			
1 to 30 days			
mg/month	3133 ± 1990	5185 ± 2414	0.02
mg/kg/month	57.2 ± 37.7	93.5 ± 44.0	0.03
31 to 60 days			
mg/month	833 ± 238	809 ± 390	0.85
mg/kg/month	14.1 ± 5.2	13.9 ± 6.1	0.92
61 to 90 days			
mg/month	700 ± 212	597 ± 218^a^	0.19
mg/kg/month	11.8 ± 4.1	10.2 ± 3.5^a^	0.25
91 to 120 days			
mg/month	562 ± 212^b^	526 ± 195^a^	0.62
mg/kg/month	9.4 ± 3.6^b^	9.1 ± 3.5^a^	0.82
151 to 180 days			
mg/month	387 ± 186^c^	423 ± 162^d^	0.61
mg/kg/month	6.2 ± 2.9^c^	7.3 ± 3.1^d^	0.39
Immunosuppressive agent, n (%)	1 (11.1%)	19 (35.1%)	0.25
**Antifibrotic agent**			
Within 1 year after AE, n (%)	2 (22.2%)	6 (11.1%)	0.31
**Additional therapy on admission**			
NIV	0 (0%)	6 (11.1%)	0.58
Antibiotics	7 (77.7%)	39 (72.2%)	> 0.99
PCP prophylaxis	9 (100%)	52 (96.2%)	> 0.99

Values are shown as actual numbers or means ± standard deviation.

^a^n = 50, ^b^n = 8, ^c^n = 6, ^d^n = 48.

*AE* acute exacerbation, *NIV* noninvasive ventilation, *PCP* pneumocystis pneumonia.

## Discussion

This study found that the total dose of corticosteroids over days 1 to 30 after the diagnosis of AE-IPF were associated with subsequent recurrence(s) of AE-IPF within 1 year after the date of admission for the first AE-IPF. A treatment that combined steroids with another immunosuppressive agent was not associated with recurrent AE. Furthermore, antifibrotic therapy administered within 1 year after AE-IPF was not associated with a subsequent recurrence. To our knowledge, this is the first study which has shown a significant association between the dose of corticosteroid used to treat a first AE-IPF and the subsequent recurrence of AE.

Corticosteroids often cause serious side effects, especially at high doses administered over a long period of time^6^. However, the initial dose of corticosteroid for an AE-IPF is usually reduced gradually because of concern about a recurrence. Our study found that only the initial dose of a corticosteroid during the first month after the onset of an AE-IPF was important in regard to preventing a recurrence of an AE. It might be possible that the initial dose of a corticosteroid can be reduced rapidly after the first month of treatment for the AE-IPF. If this can be verified, the frequency and severity of side effects associated with corticosteroid therapy could be markedly reduced.

An immunosuppressive agent tended to be more frequently used in addition to a corticosteroid for the patients who did not develop a recurrence of AE-IPF than for those who did develop a recurrence (11.1% use by patients with vs. 35.1% use by patients without), although statistical significance was not achieved. An immunosuppressive agent might be a possible treatment option not only for treating patients with an AE-IPF, but also for preventing recurrence of an AE, although further study is needed to test this hypothesis.

The antifibrotic agents nintedanib and pirfenidone are approved drugs for the treatment of IPF. Nintedanib was reported to reduce the risk of AE-IPF in an efficacy study of patients with IPF^7^. Pirfenidon has also been reported to reduce the incidence of AE after surgical resection for patients with IPF and lung cancer^8^. In our study, however, antifibrotic therapies given within 1 year after the first AE-IPF were not associated with the recurrence of an AE-IPF. Antifibrotic agents have been usually started in our institution after the patient has stabilized. Given this practice for administration of antifibrotic agents, the duration of antifibrotic therapy given to some of the study patients over the year of observation after the diagnosis of an AE-IPF might have been too short. If the observation period had been longer than 1 year, a different result might have been observed. Another possible explanation for our finding is that because a small number of patients received the agents, the effects of antifibrotics were missed. The efficacy of antifibrotic agents for reducing the risk of a recurrent AE-IPF should be investigated further.

Another important finding of the study is that a recurrence of AE-IPF is also a risk factor for the subsequent recurrence of an AE. In this study, of the patients developing a recurrence of AE-IPF after their first AE, 42.8% developed a second recurrence. This result is similar to that of the previous study, which demonstrated that of 27 patients with an AE-IPF, 7 (25.9%) had more than 1 episode of AE^9^. Among the 7, two (28.6%) had a history of 3 episodes of AE-IPF. The recurrences reportedly occurred in most patients over a short period of time (mean time to recurrence: 6 months, range 2–38 months) after the previous AE. In our present study, the mean time to recurrence of an AE-IPF was 7.7 (range 2–12) months, which was similar to the previous report. There may be a phenotypic patient with IPF who develops frequent exacerbations.

This study has limitations. First, this was a single-center study with a relatively small number of patients. Second, the study was conducted in a retrospective fashion. A multicenter study employing prospective enrollment of a larger number of patients is needed to confirm the results of our small study. Third, our study might have included patients with pulmonary infections. Major efforts were made to exclude patients with obvious pulmonary infections. However, distinguishing clearly between an AE-IPF and pulmonary infection is sometimes quite difficult. The recent international working group report of the American Thoracic Society (ATS) proposed 2 types of AE-IPF, idiopathic versus triggered AE-IPF^4^. A triggered AE includes an AE occurring after an infection, procedure, or surgery; or an AE associated with drug toxicity or aspiration. Distinguishing between infection-triggered AE and simple pulmonary infection is sometimes impossible. Fourth, data at the discharge from the chest HRCT, pulmonary function testing, the six-minute walk test, and echocardiography could not be used for the analysis, because they were not performed in most cases.

In conclusion, when comparing IPF patients hospitalized for their first AE, which was treated by steroids, who were stratified according to whether or not they developed an early subsequent recurrence of an AE, a higher total dose of corticosteroids administered over days 1 to 30 after admission was associated with a lower risk of early recurrence of a subsequent AE-IPF.

## Methods

### Patients

A retrospective review was performed of the files of consecutive patients with IPF who were admitted to the Kindai University Hospital for their first AE between January 2008 and December 2018 and were discharged alive. Whether or not the patient developed a recurrent AE within 1 year from the date of onset of the first AE was determined. The diagnosis of AE-IPF was made according to the recent international working group report of the ATS, as follows: 1) a previous or concurrent diagnosis of IPF; 2) acute worsening or development of dyspnea typically of less than 1-month’s duration; 3) computed tomography study of the lungs showing new bilateral ground-glass opacities and/or consolidations superimposed on a background pattern consistent with UIP; 4) deterioration not fully explained by cardiac failure or fluid overload^4^. The diagnosis of IPF was made according to the criteria used in the INPULSIS trial^7^. If a surgical lung biopsy was not performed, a HRCT study was required, which showed the following findings: honeycombing and/or a combination of reticular abnormalities and traction bronchiectasis consistent with fibrosis with basal and peripheral predominance and without atypical features of UIP^7^. The study protocol was approved by the ethics committee of the Kindai University Faculty of Medicine (No. 31-228). Informed consent was waived, because this study was based on a retrospective analysis of case records from our university hospital. All methods were performed in accordance with the relevant guidelines and regulations (Declaration of Helsinki).

### Baseline pulmonary function testing

Pulmonary function test results that were obtained during the stable phase of the disease within 1 year prior to the AE were adopted as baseline values. Pulmonary function testing was performed by the CHESTAC-8800 system (Chest, Tokyo, Japan), according to the standards proposed by the European Respiratory Society^10,11^.

### Data collection

The following baseline characteristics of the patients with AE-IPF were determined, as follows: gender, age, body mass index, smoking status, long-term oxygen therapy, and treatments for IPF. Admission and discharge results of routine blood tests were evaluated.

### Evaluation of the treatments for AE-IPF

The total dose and the total dose per body weight (kg) of corticosteroids administered over each month after the initiation of steroid therapy were evaluated. Whether or not immunosuppressive agents were used with corticosteroids was assessed. Use of an antifibrotic agent within 1 year after the day of admission for the first AE was also examined.

### Recurrence of AE-IPF

The recurrence of an AE-IPF within 1 year after the date of admission for the first AE-IPF was reviewed in each patient. The time to recurrence was defined as the number of days from the date of admission for the first AE-IPF until the date of admission for the recurrent AE.

### Statistical analysis

Continuous variables were expressed as means ± standard deviation (SD), and categorical variables were expressed as actual numbers. The unpaired *t*-test was used for continuous data and the χ^2^ test or Fisher exact test was used for categorical data between 2 groups. Multivariable analysis with logistic regression was used to identify the risk of AE-IPF recurrence. In this analysis, variables with *p* < 0.2 on univariate analysis and variables historically considered to be important in IPF were included as independent variables in the multivariable analysis. A *p* value of < 0.05 was considered statistically significant. Analyses were performed with Statflex, ver.6 software (Artech, CO., Ltd., Osaka, Japan).

## Data Availability

All data are available if requested.
